# 

**Published:** 1880-10

**Authors:** 


### CONTENTS.

COMMUNICATIONS.

Diagnosis of Malignant Tumors of the Upper Jaw in Youth.......... 425
General Care of the Mouth for Patients under Twenty Years of Age... 432
Materia Medica and Therapeutics.......................... 438
What Kills.......................................... 443
Report of the discussions before the Kentucky State Dental Association 444
Preparation for Medical and Dental Study.................. 459
Compensation for Dental Services.......................... 464

EDITORIAL.

Treatment of Sensitive Dentine............................ 466
Diversion.......................................... 468
Why Is It?.......................................... 470
Engagement Book.......................................... 470
Absence.......................................... 470

